# Disturbed diastolic left ventricular inflow vortex ring formation in patients with corrected atrioventricular septal defect: quantitative three-dimensional vortex core analysis from 4DFlow MRI

**DOI:** 10.1186/1532-429X-17-S1-O4

**Published:** 2015-02-03

**Authors:** Mohammed SM ElBaz, Emmeline Calkoen, Arno Roest, Jos J Westenberg, Rob J van der Geest

**Affiliations:** 1 Pediatric cardiology, Leiden University Medical Center, Leiden, Netherlands; 2 Division of Image Processing, Department of Radiology, Leiden University Medical Center, Leiden, Netherlands

## Background

Vortex formation in the left ventricle (LV) is suggested to contribute to efficient blood pumping and altered vortex formation is associated with diastolic dysfunction. Patients after atrioventricular septal defect (AVSD) correction present abnormalities in valve morphology and subsequently develop altered inflow patterns (Calkoen et al. JMRI 2014), which may disturb normal vortex formation. We aimed to analyze vortex ring formation in AVSD-corrected patients compared to healthy controls and to evaluate association with inflow angle.

## Methods

23 patients (age 20±8 years) and 23 healthy controls (age 19±8 years) were included. Whole-heart 4DFlow MRI was performed on 3T MRI with free breathing, three-directional velocity encoding of 150cm/s in all directions, spatial resolution 2.3×2.3×3.0-4.2mm^3^ and 30 phases reconstructed over one cardiac cycle. The Lambda2 method was used to depict the cores of three-dimensional (3D) vortex ring structures in the velocity field in the LV at the phases of peak early filling (E-peak) and peak late filling (A-peak) (2). Subsequently, the 3D location of the vortex ring center was defined using the circumferential (C), longitudinal (L) and radial (R) coordinates as well as vortex orientation relative to the LV long-axis (Elbaz et al. JCMR 2014). The circularity index (CI) was calculated to quantify the shape of the vortex ring. Measured parameters of controls and patients were compared using chi-square tests and non-parametric t-tests. In patients, Pearson's correlation between vortex orientation and previously described inflow angle (Calkoen et al. JMRI 2014) was analyzed.

## Results

A distinct vortex ring core at E-peak was observed in all controls and in 18 (78%) patients (p=0.025). An A-peak vortex ring core was detected in 20 (87%) controls and 11 (49%) patients (p=0.014). Quantitative characteristics of detected vortex ring cores at E-peak and A-peak are presented in Table 1. The larger value in R-coordinate observed in patients indicates a more lateral position of the vortex core center in patients during E-peak and A-peak. In patients a less circular shape of the vortex core was observed compared to controls. Orientation of the vortex core in patients showed a wide range (13 - 125 degrees). In patients the correlation between vortex orientation and inflow angle was r=0.5 (p=0.06) at E-peak and r= 0.7 (p=0.01) at A-peak.

**Table 1:** Quantitative vortex core characteristics at E-peak and A-peak in controls and patients.

		Controls (N=23)	Patients (N=18)	P
E-peak	C	89 ± 23	85 ± 20	0.603
	
	L	0.19 ± 0.04	0.16 ± 0.06	0.125
	
	R	0.26 ± 0.06	0.40 ± 0.11	<0.001
	
	Orientation	71 ± 8	61 ± 32	0.215
	
	Circularity Index	0.78 ± 0.07	0.68 ± 0.16	0.018

		Controls (N=20)	Patients (N=11)	

A-peak	C	101 ± 23	98 ± 19	0.706
	
	L	0.14 ± 0.05	0.15 ± 0.07	0.755
	
	R	0.20 ± 0.09	0.45 ± 0.20	0.002
	
	Orientation	74 ± 4	54 ± 24	0.019
	
	Circularity Index	0.66 ± 0.09	0.55 ± 0.11	0.009

*indicates significant different between patients and controls with P<0.05, ** indicates p<0.01. Heart rate was similar in controls (N=23, 72 ± 13 bpm) and patients ( N=18, 77 ± 10 bpm, p=0.30). No significant difference was observed in Left ventricular end diastolic volume between controls (N=23, 139 ± 41 mL) and patients (N=18, 153 ± 36 mL). Ejection fraction was significantly smaller in patients (N = 18, 56 ± 7%) compared to controls (N = 23, 61 ± 5%), p = 0.005.

## Conclusions

Presence and formation of vortex ring core is aberrant in patients with corrected AVSD compared to healthy controls. Different position and circularity of the vortex cores are possibly due to abnormalities in the left atrioventricular valve. Altered vortex orientation is correlated with patients' lateral inflow. Future studies are required to investigate the effect of absence or altered vortex ring formation on energy loss.

## Funding

E.E. Calkoen is financially supported by a grant from the Willem-Alexander Kinder- en Jeugdfonds, M.S. ElBaz and J.J.M. Westenberg are financially supported by a grant from the Dutch Technology Foundation (STW).

**Figure 1:**
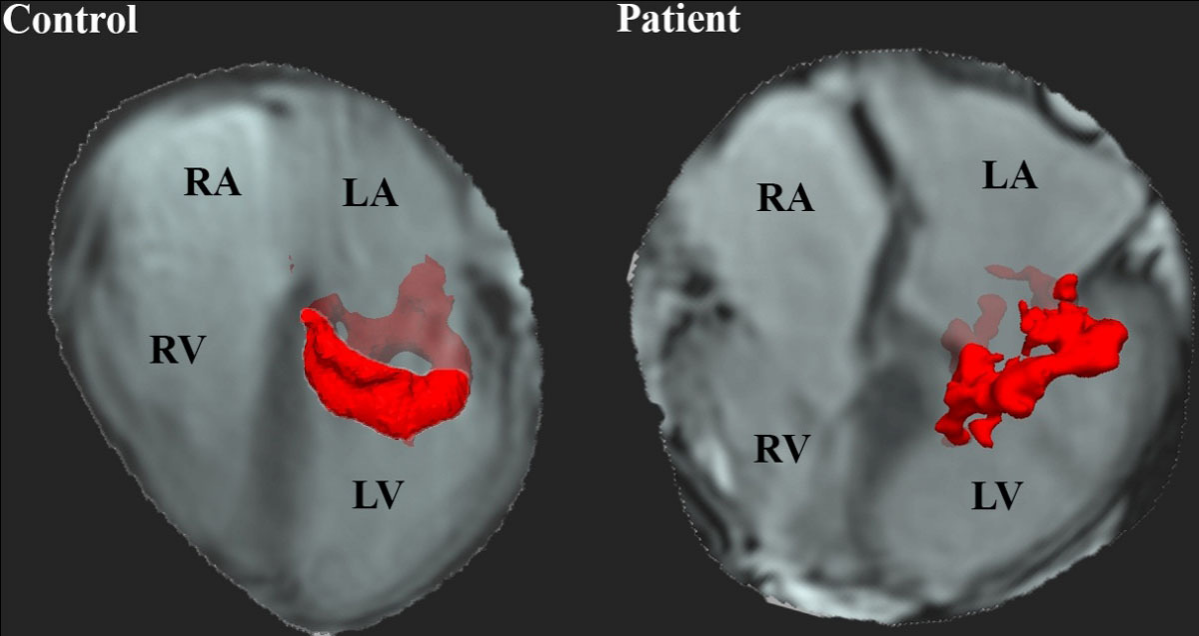
Vortex ring core in a control and a patient during E-peak. In the patient a more tilted (orientation 37 degrees) and more lateral positioned (R = 0.39) vortex was observed. RA = right atrium, RV = right ventricle, LA = left atrium, LV = left ventricle.

